# Association between fatality rate of COVID-19 and selenium deficiency in China

**DOI:** 10.1186/s12879-021-06167-8

**Published:** 2021-05-19

**Authors:** Hai-Yang Zhang, An-Ran Zhang, Qing-Bin Lu, Xiao-Ai Zhang, Zhi-Jie Zhang, Xiu-Gang Guan, Tian-Le Che, Yang Yang, Hao Li, Wei Liu, Li-Qun Fang

**Affiliations:** 1 State Key Laboratory of Pathogen and Biosecurity, Beijing Institute of Microbiology and Epidemiology, 20 Dong-Da Street, Fengtai District, Beijing, 100071 People’s Republic of China; 2 School of Public Health, Cheeloo College of Medicine, Shandong University, 44 West Wenhua Road, Jinan, Shandong 250012 People’s Republic of China; 3 College of Public Health and Health Professions, Emerging Pathogens Institute, University of Florida, Gainesville, Florida, 32610 USA; 4 School of Public Health, Peking University, Beijing, 430071 People’s Republic of China; 5 Department of Epidemiology and Health Statistics, School of Public Health, Fudan University, Shanghai, People’s Republic of China

**Keywords:** COVID-19, Case fatality rate, Selenium, Micronutrient, China

## Abstract

**Background:**

COVID-19 has impacted populations around the world, with the fatality rate varying dramatically across countries. Selenium, as one of the important micronutrients implicated in viral infections, was suggested to play roles.

**Methods:**

An ecological study was performed to assess the association between the COVID-19 related fatality and the selenium content both from crops and topsoil, in China.

**Results:**

Totally, 14,045 COVID-19 cases were reported from 147 cities during 8 December 2019–13 December 2020 were included. Based on selenium content in crops, the case fatality rates (CFRs) gradually increased from 1.17% in non-selenium-deficient areas, to 1.28% in moderate-selenium-deficient areas, and further to 3.16% in severe-selenium-deficient areas (*P* = 0.002). Based on selenium content in topsoil, the CFRs gradually increased from 0.76% in non-selenium-deficient areas, to 1.70% in moderate-selenium-deficient areas, and further to 1.85% in severe-selenium-deficient areas (*P* < 0.001). The zero-inflated negative binomial regression model showed a significantly higher fatality risk in cities with severe-selenium-deficient selenium content in crops than non-selenium-deficient cities, with incidence rate ratio (IRR) of 3.88 (95% CIs: 1.21–12.52), which was further confirmed by regression fitting the association between CFR of COVID-19 and selenium content in topsoil, with the IRR of 2.38 (95% CIs: 1.14–4.98) for moderate-selenium-deficient cities and 3.06 (1.49–6.27) for severe-selenium-deficient cities.

**Conclusions:**

Regional selenium deficiency might be related to an increased CFR of COVID-19. Future studies are needed to explore the associations between selenium status and disease outcome at individual-level.

## Background

The recent outbreak of coronavirus disease 2019 (COVID-19) caused by severe acute respiratory syndrome coronavirus 2 (SARS-CoV-2) has turned the world into chaos with its ominously high rate of transmissions. To the end of 2020, the pandemic has resulted in more than 1,618,374 deaths, however, with the case fatality rate (CFR) varying dramatically across countries [1]. Currently, there is no specific treatment with proven therapy for COVID-19, especially for those with severe disease. Although some clinical studies have provided a reference for treating of patients, which helps some patients recover quickly, such as remdesivir [2], the efficacy of these drugs remained highly controversial. The factors that might contribute to the disease outcome had been intensively explored, which included age, gender, underlying conditions and others. For these known factors, the functional mechanisms underlying their role in clinical phenotype was suggested to be orchestrated by the host immune response.

Immune support by micronutrients, including vitamins A, D, C, E, B6, and B12, folate, zinc, iron, copper, and selenium, had long been established to play pivotal role in the defense against infectious diseases [3, 4]. Among all the micronutrients implicated in viral infection, selenium is of high importance for human health and particularly for a well-balanced immune response, mostly through its function in antioxidant defense, redox signaling, and redox homeostasis [5–8]. As demonstrated in previous studies, its deficiency has been related to higher susceptibility to RNA viral infections and more severe disease outcome [6, 9, 10]. Selenium status not only affect the host immune response but also alter the viral pathogen itself [6, 11, 12]. The deficiency of selenium leads to oxidative stress in the host, thus alter the genome of RNA viruses in the way that a normally benign or mildly pathogenic virus becomes highly virulent in the selenium deficient and oxidatively stressed host, for instance, human immunodeficiency virus (HIV), coxsackie virus, and influenza virus [7, 11, 13, 14]. The differences in the baseline immune capabilities due to intake of selenium might influence the occurrence and outcome of multiple viral infectious diseases [5, 6, 15, 16], although observational studies associations between selenium intake and these diseases have been inconsistently shown in several studies [17, 18]. Based on these previous knowledges, it is justified to hypothesize that the selenium status might as well impact the infection of SARS-CoV-2.

The intake of selenium varies worldwide, and China is known to be one of the most severely selenium deficient countries in the world, with a wide range of selenium level that differs from the lowest to the highest in the world. Considering that fatality rate of COVID-19 also varied across different afflicted regions in China [19], we wondered if there might be a relationship between disease severity of COVID-19 and selenium level [20]. Here we applied an ecological study to evaluate the association between COVID-19 related fatality and the selenium content in crops and topsoil at the population level in China.

## Materials and methods

### Data collection on COVID-19 patients and selenium level

The data on confirmed COVID-19 cases and deceased cases at the city level in the mainland of China reported during 8 December 2019–13 December 2020 were obtained from the DXY website, a nongovernment website that provided daily updates of the reports from official sources, mainly involving websites of the health commissions of each province, municipality, or city [21]. The CFRs at the city level were calculated by dividing the observed number of deaths by the number of confirmed cases. As we know, Hubei Province, as the first epicenter of COVID-19 outbreak, suffered a great chaos and shortage of medical and health source at the early stages of the first wave of COVID-19 epidemic, which has not occurred in any other provinces in China. The crude case fatality rate in Hubei Province were 7.3 times of that in the combination of all other provinces at the first wave of the epidemic [22]. Hubei Province was excluded from the current analysis to avoid biasing the association between fatality rate of COVID-19 and selenium deficiency due to the influence of the shortage of medical and health source in Hubei Province. Cities that reported less than 20 cases were also excluded to avoid the bias from small sample size. Cases imported from abroad were excluded from the analysis because their clinical phenotype was largely not influenced by the selenium content in China.

This study was approved by the institutional review board of the Beijing Institute of Microbiology and Epidemiology (Beijing, China). All data were collected from publicly available sources. Data were de-identified, and informed consent was waived.

Data on social-demographic co-variables, possibly related to the mortality of COVID-19 mortality, including population density, proportion of population over 60 years old, and gross domestic product (GDP) per capita at the city level were collected from the website of National Bureau of Statistics of China (most recent update in the year of 2017) (www.stats.gov.cn). Additionally, three key medical access related variables including number of hospitals, number of hospital beds, and number of clinical staffs per 1000 people, were also collected from National Bureau of Statistics of China (www.stats.gov.cn). Selenium content measured from crops (including corn, barley, rice, sorghum, millet, potatoes, broad beans, wheat, etc.) at the county level [23] and from topsoil at sampling site level [24], were used for the current analysis. Both data were obtained from the Chinese Academy of Agricultural Sciences.

Then selenium content of crops for each city were calculated by averaging the selenium content at county level. The selenium content in topsoil for each city was defined as the weighted average of area-based selenium content. All cites were classified into 3 categories: non-selenium-deficient areas (> 0.06 ppm), moderate-selenium-deficient areas (0.03–0.06 ppm), and severe-selenium-deficient areas (< 0.03 ppm) according to selenium content in crops, and were grouped into non-selenium-deficient areas (concentration > 0.31 mg/kg), moderate-selenium-deficient areas (0.18–0.31 mg/kg), and severe-selenium-deficient areas (< 0.18 mg/kg) according to selenium content in topsoil.

### Geographical analysis of COVID-19 mortality

Each COVID-19 case was geo-referenced to the corresponding polygons of the China digital map through the linkage of the 4-digit city geo-code. The average CFR that was calculated at city level was overlapped on the selenium concentration of crops and topsoil to create a thematic map by using ArcGIS 10.7 (Environmental Systems Research Institute Inc., Redlands, CA, USA). The COVID-19 related fatality rates were compared across different selenium levels by chi-squared test. The Zero-inflated negative binomial regressions were applied to estimate the relationship between CFR of COVID-19 and selenium deficiency by using “zeroinfl” function in R package “pscl”. The number of COVID-19 deaths per city was set as the outcome variable, and the total case number was included as an “offset term (in log-transformed form)” included in model for each city. The population density, GDP per capita, proportion of population over 60 years old, number of hospitals, number of hospital beds, and number of clinical staffs per 1000 people were included as co-variables. The incidence rate ratio (IRR) in response to the change of each variable by a given amount (e.g.,100 persons per km^2^ for population density, 10 thousand Yuan per person for GDP per capita, and 1% for proportion of population over 60 years old) was used to determine its impact on CFR. The 95% confidence intervals (CIs) and *P* value were estimated after correcting for over dispersion, due to spatial clustering patterns and zero death in some cities [25]. The univariate analyses were separately performed to examine the effect of each variable, based on which co-variables with a *P* < 0.20 were further included for the multivariate analysis. A *P* value of < 0.05 was considered statistically significant. Statistical analysis was done using R version 3.6.3.

## Results

### Geographical analysis of COVID-19 fatality

From 8 December 2019 to 13 December 2020, a total of 15,434 confirmed COVID-19 cases were reported from 312 cities outside Hubei Province. Totally, 64 cities had reported 120 cases outside Hubei Province, leading to an overall CFR of 0.78% (120/15,385). For the cities with deaths, the median fatality rate was 3.12% (IQR: 1.38–6.26%). A total of 147 cities each reporting over 20 cases were included in the current analysis. In these cities, 91% (14,045) of total cases and 85.8% (103) of total mortality from COVID-19 in China had been reported.

**Fig. 1:**
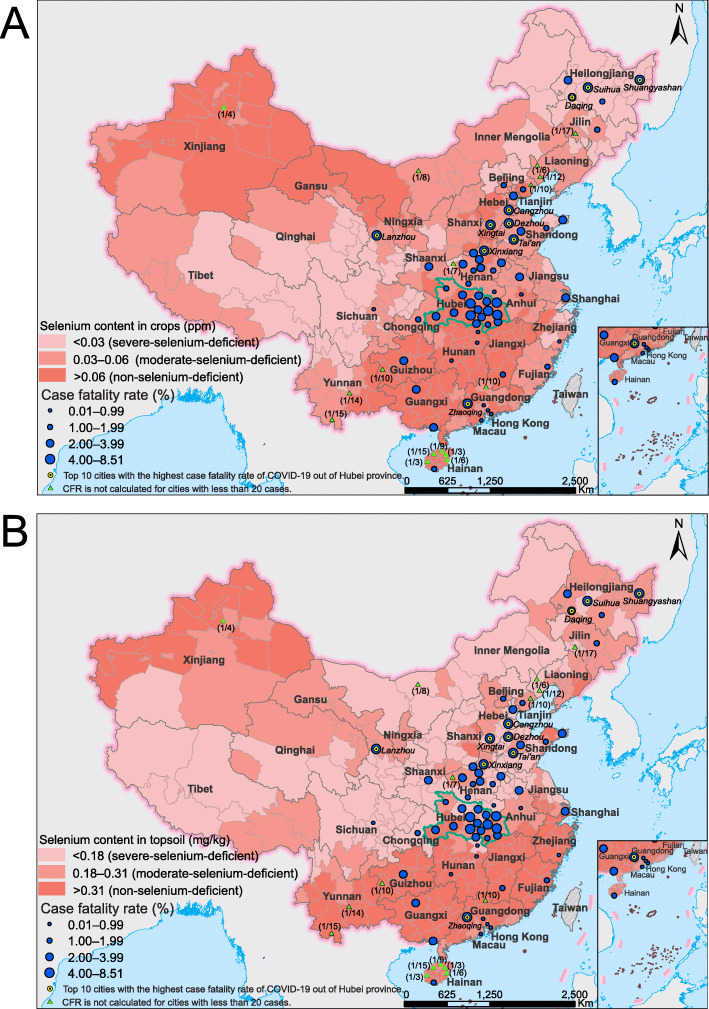
The case fatality rate of COVID-19 and selenium deficiency at the city level based on selenium content in crops (Panel **a**) and topsoil (Panel **b**) in the mainland of China. The background color of the map represents the selenium level at the city level. The dots display the average fatality rate of COVID-19 at the city level. The average fatality rate was only shown for cities reporting least one death. The fatality rate of cities with < 20 deaths are indicated in brackets

The CFR of COVID-19 on the map of selenium content in crops at the city level (Fig. 1a). Among the studied 147 cities, 75 (51.0%) and 22 (15.0%) were grouped into moderate and severe selenium deficient areas based on selenium content in crops, respectively. This is largely consistent with the grouping based on the selenium content in topsoil (Spearman correlation coefficient of 0.46, *p* < 0.001). Among the top 10 cities with the highest CFRs (3.70–8.51%), four cities were grouped as severe-selenium-deficient regions and five were moderate-selenium-deficient regions (Table 1, Fig. 1a). Most of the hotspots of highest CFRs of COVID-19 fell within the selenium-deficient belt [26], located in northeastern and central areas of the mainland of China, respectively. Notably, in Heilongjiang Province in northeastern China, significantly higher CFRs ranging from 1.52 to 8.51% were observed from the cities with the most severe-selenium-deficiency in China [27]. For other provinces, such as in Shandong, Hebei, and Henan provinces, neighboring cities with comparable socioeconomic level, had also displayed CFRs with high variance, which were correlated with the extent of selenium deficiency (Fig. 1a).

**Table 1:** Top 10 cities with the highest case fatality rates of COVID-19 in China

City^a^	No. of cases	Case fatality rate (%)	Selenium feed level^**b**^	Selenium soil level^**c**^
Suihua	47	8.51	severe-selenium-deficient	severe-selenium-deficient
Cangzhou	49	6.12	moderate-selenium-deficient	severe-selenium-deficient
Shuangyashan	52	5.77	severe-selenium-deficient	moderate-selenium-deficient
Taian	35	5.71	severe-selenium-deficient	severe-selenium-deficient
Lanzhou	36	5.56	moderate-selenium-deficient	moderate-selenium-deficient
Dezhou	37	5.41	moderate-selenium-deficient	severe-selenium-deficient
Xinxiang	57	5.26	moderate-selenium-deficient	severe-selenium-deficient
Zhaoqing	20	5.00	non-selenium-deficient	non-selenium-deficient
Xingtai	23	4.35	moderate-selenium-deficient	severe-selenium-deficient
Daqing	27	3.70	severe-selenium-deficient	moderate-selenium-deficient

^a^We included only 147 cities from which at least 20 cases were reported

^b^Selenium content in crops: Severe-selenium-deficient areas indicate selenium content in crops: < 0.03 ppm (ppm); Moderate-selenium-deficient areas indicate selenium content in crops: 0.03–0.06 ppm (ppm); non-selenium-deficient indicate selenium content in crops: > 0.06 ppm

^c^Selenium content in topsoil: Deficient areas indicate selenium content in topsoil: < 0.18 mg/kg; Moderate-selenium-deficient areas indicate selenium content in topsoil: 0.18–0.31 mg/kg; non-selenium-deficient indicate selenium content in topsoil: > 0.31 mg/kg

The CFR of COVID-19 was further overlapped on the map of selenium content in topsoil (Fig. 1b). In line with results from selenium content in crops, nine among the top 10 cities with the highest CFRs were selenium deficient, including six severe-selenium-deficient regions and three moderate-selenium-deficient regions (Table 1).

### Correlation analysis between COVID-19 fatality and selenium deficiency

The overall CFRs of COVID-19 for the analyzed cities were inversely related to the selenium content in crops, which gradually increased from 1.17% in non-selenium-deficient areas, to 1.28% in moderate-selenium-deficient areas, and further to 3.16% in severe-selenium-deficient areas (*p* = 0.002, Table 2). The zero-inflated negative binomial regression model showed a significantly higher risk of mortality conferred from severe-selenium-deficient cities, with incidence rate ratio (IRR) of 3.88 (95% CIs: 1.21–12.52), in comparison with non-deficiency (*P* = 0.023; Table 2). No statistical difference of CFR due to COVID-19 was shown between moderate-selenium-deficient cities and non-selenium-deficient cities (IRR: 1.92, 95% CIs: 1.00–3.69, *P* = 0.051).

**Table 2:** Association between case fatality rate of COVID-19 and the selenium level in China

City selenium level^a^	Crude CFR (%) ^**b**^	***P*** value^**b**^	Zero-inflated negative binomial regression^**d**^	
			IRR (95% CIs) ^**c**^	***P*** value
**Selenium content in crops (ppm)**				
> 0.06 (non-selenium-deficient areas)	1.17	0.002	1	–
0.03–0.06 (moderate-selenium-deficient areas)	1.28		1.92 (1.00–3.69)	0.051
< 0.03 (severe-selenium-deficient areas)	3.16		3.88 (1.21–12.52)	0.023
**Selenium content in topsoil (mg/kg)**				
> 0.31 (non-selenium-deficient areas)	0.76	< 0.001	1	–
0.18–0.31 (moderate-selenium-deficient areas)	1.70		2.38 (1.14–4.98)	0.021
< 0.18 (severe-selenium-deficient areas)	1.85		3.06 (1.49–6.27)	0.002

^a^We included only 147 cities from which at least 20 cases were reported

^b^The crude case fatality rates (CFRs) were calculated by dividing the number of deaths by the number of cases within the region. The *P* values were calculated by chi-square tests

^c^IRR (95% CIs): incidence rate ratio with 95% confidence intervals

^d^The Zero-inflated negative binomial regression were used for evaluating the association between the selenium level and case fatality rate at the city level. Only IRR (95%CIs) of count model (negative binomial with “log” link) was shown in the table, and the multivariate analysis had the same results with the univariate analysis due to non-significant effects of demographic, social and medical access related covariates, including population density, GDP per capita, proportion of population over 60 years old, number of hospitals, number of hospital beds, and number of clinical staffs per 1000 people﻿ on the case fatality rate for COVID-19

A more robust effect was observed when selenium content in topsoil was used for correlation analysis. The crude CFRs from non-selenium-deficient to severe-selenium-deficient areas were 0.76%, 1.70% and 1.85% respectively (*p* < 0.001, Table 2). The result of zero-inflated negative binomial regression model revealed significantly increased CFR that was observed from both moderate-selenium-deficient and severe-selenium-deficient cities, with the IRR (95% CIs) estimated to be 2.38 (1.14–4.98) and 3.06 (1.49–6.27), respectively, when using non-selenium-deficient cities as reference. For both models built on different selenium contents, no significant effect on CFR of COVID-19 was observed from other demographic, social or medical access related variables, including population density, GDP per capita, and proportion of population over 60 years old, number of hospitals, number of hospital beds, and number of clinical staffs per 1000 people (data not shown).

## Discussion

In this study, a statistically significant ecological association between CFR of COVID-19 and selenium deficiency, measured from both crops and topsoil, was found in the mainland of China. In comparison with previous study that reported a significant association between cure rate and background selenium status in cities outside Hubei [20], our study had the advantage that most recent updated data were used for analysis. The current data on intake of selenium were obtained based on the most extensive investigation of selenium content in crops that covered all 2922 counties, and 4095 investigation sites of selenium content in topsoil. The highly credible fatality data in China was accessible as the epidemic has been largely brought to an end, with highly sporadic new cases and no new death reported for a long duration, also giving an optimal opportunity to make fatality related analysis. A current clinical study performed in German verified the association between Se and clinical outcomes of COVID-19, where a pronounced deficit in total serum Se was displayed in SARS-COV-2 infected cases, with Se deficiency more severe in the non-survivors as compared with survivors of COVID-19 [28].

The potential mechanism how the selenium deficiency can lead to a more severe infection is complex. Most of biological functions of selenium are exerted through its incorporation as a rare amino acid selenocysteine in the essential family of selenoproteins [5, 29]. At least 25 selenoproteins have been identified in humans [29], most of which are involved in a redox reaction [8]. Glutathione peroxidases, the major components of the human antioxidant defense, play a critical role in redox signaling and other immune responses in acute stress conditions [8, 12]. This has been evidenced by an obvious correlation between low selenium and worse clinical outcomes in critically ill patients [30]. Interestingly a recent metabolomic profiling of SARS-COV-2 infected VeroFM cells had revealed the most pronounced upregulation of glutathione and pyrimidine metabolism, which were responsible for elimination of reactive oxygen species [31], implying the induction of antioxidant defense against SARS-COV-2 infection, and thus indicating a potential role of biological activities of selenium during this process. Besides, like other RNA viruses including HIV and Ebola virus, SARS coronaviruses could probably use host cellular selenium to complete the expression of the viral selenopeoteins [32, 33], thus, a selenium deficient status in the infected host might be induced during the acute phase of illness when the virus intensely replicated. A recent review suggested that redox-active selenium species formed at high selenium intake might hypothetically inhibit SARS-CoV-2 proteases. Thus, by interfering with the human selenoprotein system, SARS-CoV-2 could evade an adequate host response [34].

On the other hand, selenium might affect the blood coagulation and thrombosis formation in the form of sodium selenite that could decrease thromboxane A2 (TXA2) formation that plays a key role in blood platelet activation and aggregation [35, 36]. It is well documented from a number of hemorrhagic fever viruses, including Hantavirus and Ebola virus that selenium might be involved in the hemorrhagic manifestations by affecting the prostacyclin/TXA2 ratio and thus blood clotting [37]. This mechanism could support the association between selenium deficiency and more coagulation dysfunction in COVID-19, i.e., thrombocytopenia, prolonged APTT and PT, as well as increased DIC development, according to the current findings. All these indicators are critical factors in determining the disease severity in COVID-19 [38], thus providing potential mechanism how selenium could be involved in this process. A most recent study, based on a linear association between selenium deficiency with hypoglycemia in healthy adults, also supported the role of adequate Se supply for glucose homeostasis in human subjects [39]. This newly identified relationship may be of relevance for the course of severe complications in COVID-19 with mortality risk. Recognition of these mechanisms by which selenium might potentially benefit COVID-19 patients provides a rationale for selenium supplementation in SARS-CoV-2 infection. Existing data on the effect from daily selenium supplementation to treat viral infection, most notably with respect to HIV and influenza A virus infections, are heterogeneous [16]. In one clinical study, although selenium supplementation delayed CD4 decline in HIV-infected patients, no evidence of suppressing or reducing HIV viral load was seen [40]. Inconsistent results of benefits with routine multiple micronutrient supplementation in HIV infection was noticed from numerous clinical trials [41]. However, these findings should not be interpreted as a reason to deny micronutrient supplements for people with known specific deficiencies or with lower diet level under the recommended allowance. Particularly for the eldly, who had high odds of progressing into fatal COVID and were more likely to be with selenium deficiency, might gain some benefit from prophylactic micronutrition supplementation.

The study was subject to several limitations. First, selenium status in the population could not be fully represented by the selenium concentration measured from local crops or topsoil, since the supplies of food were not restricted to local sources and dietary habits also played key role in effective selenium intake [42]. Besides, the effect of selenium deficiency might be confounded by several other insufficient dietary nutrients such as vitamins, Ca, Fe, Zn and I [43], and the migration bias related with transport of crops or routes of foods might also exist [44]. However, considering that almost all provinces in China except for Beijing and Shanghai had been self-sufficient in grain and vegetables according to the production and consumption data of grain and vegetables of them from China Statistical Yearbook 2020 of National Bureau of Statistics (http://www.stats.gov.cn), it’s reasonable to assume that local food and drinking water are the major source of selenium intake, thus causing minor bias in eliciting the association. Additionally, given the persistent travel restriction during the intense COVID-19 epidemic, the migration bias due to transport of crops or routes of foods could be minimized. Second, the nationwide data on selenium concentration in crops or topsoil are old. As we known, the nationwide selenium data are not updated in recent decades due to the large amount of work for conducting a nationwide survey of selenium concentration in crops or topsoil. Besides, bioavailability of selenium in soil (such as pH, humic matter content), which could affect the process of selenium in topsoil entering the food chain [45], was not taken into account in the analysis. At last, our data of selenium concentration in crops or topsoil were categorical variable which limited us for further assessing the potential threshold or nonlinear relation effects. Given the toxic effect of excess selenium, it should be cautious about extrapolation of current results. Despite of current findings, the role of selenium in COVID-19 needs to be replicated in other selenium deficient countries and further validated at individual-level. However, when the results of ecological research are applied to the individual level, ecological fallacies should be alerted. Thus, even though previous evidences and the current study supported the risk of selenium deficiency [46, 47], additional experimental studies are warranted to explore possible mechanisms of selenium deficiency in causing death risk of COVID-19. Before selenium supplementation could be recommended for patients with SARS-CoV-2 infection in severe selenium-deficient areas, the necessary caution of avoiding selenium toxicity should be raised [48].

## Data Availability

The dataset analyzed during the current study is available from the corresponding author on reasonable request.

## Abbreviation

CFR: Case fatality rate
